# Vaccination in the Light of the Royal British Commission

**Published:** 1899-05

**Authors:** M. R. Leverson

**Affiliations:** Fort Hamilton, L. I., N. Y.


### VACCINATION IN THE LIGHT OF THE ROYAL BRITISH COMMISSION.

M. R. Leverson, M. D., Fort Hamilton, L. I., N. Y.

(Continued from April No., page 173.)

Q. 10,319. The chairman objects to the analysis of the petition, saying, "We want the report of the German Commission." But Mr. Tebb replies that the petition recites the cases which induced the German Commission to make their report—the fundamentals of the case.

Q. 10,320 (Chairman). "All you give is the assertion made in the petition presented to the Reichstag, but we cannot trust to assertions made upon either side simply."

Mr. Tebb points out that being denied information by the Berlin Board of Health it was the best they could do. The objection of the chairman seems to have been maintained, though the report does not so state. Mr. Tebb's next point is the inequality in the operation and administration of the vaccination laws and how, generally, the rich are able to evade all but a nominal compliance with them.

[The same is the case, but even to a greater degree in the United States.—Ed.]

Qs. 10,321-2 describe the cruelty exercised in some cases in their execution.

Q. 10,323 likens the pursuit of English parents seeking to escape with their children from the public vaccinator to the escape of slaves from the Southern States and their pursuit under the fugitive slave law. He had personal experience as to both, and he says: "I solemnly declare I never heard more pitiful, cruel, and heartbreaking experiences than I have listened to again and again from the lips of English mothers when detailing the intolerable wrongs done to themselves and children under the operation of the vaccination laws." [Alas for America! The maternal love which leads English mothers to seek to protect their children from the cruel lancet of the blood poisoner at the cost of so much suffering to themselves, seems wholly wanting in those States of the Union where vaccination is enforced by the denial of education to the unvaccinated.—M. R. L.]

Mr. Tebb proceeds (Q. 10,233). In reply to Q. 9,532, by Mr. Meadows White, Mr. Tebb stated that "Medical journals were generally reluctant to publish details of vaccinal disasters or anything adverse to the interests of vaccination. From that opinion there was a strong expression of dissent. It will not, therefore, be inappropriate if I mention that a diligent search in the columns of *The Lancet* and *British Medical Journal* have failed to discover any reference to the disasters at Selns, Rügen, Villefranche, Department de l'Oise, Motte aux Bois, or Elberfeld. There is only one brief allusion to the disaster at Algiers in *The Lancet*, which is rather to discredit the occurrence. No report of the results of the inquiry into vaccination by the Exeter Hall Committee under the presidency of Dr. Charles Drysdale, in 1883, has appeared, nor is there any reference to the various official reports in the Sandwich Islands, British Guiana, and Trindad, or to the cases mentioned in Dr. John D. Hillis' work on leprosy in British Guiana, admitting the connection between vaccination and leprosy, or to the disclosures in Professor Fournier's recent work. On the other hand, the two remarkable cases contributed by Professor W. T. Gairdner, of invaccinated leprosy, appeared in the *British Medical Journal* of June 11th, 1887, which contains an apology for producing them though some years after the tragic occurrences." Mr. Tebb mentions several further instances, both of medical journals, not publishing accounts of disasters, even refusing advertisements of anti-vaccination publications containing replies to pro-vaccination literature. Names *The Student's Journal and Hospital Gazette* as an honorable exception. His last point is "Official promises and performance." He handed in a table which exhibited the results of vaccination as he knows them from twenty-two years of inquiry and experience, contrasted with the statements made again and again in the medical journals, in Parliamentary returns, in the rescripts of the Local Government Board, and by the pro-vaccinal press. He quoted from a document known as the Epidemiological Society's Report, on which Lord Lyttleton based his argument for a compulsory law in England from pp. 2 and 5, as follows: "We are ourselves satisfied, and it is the concurrent and unanimous testimony of nearly 2,000 medical men with whom, as we have already stated, we have been in correspondence, that vaccination is a perfectly safe and efficient prophylactic against this disease (small-pox)." *The Lancet*, May 30th, 1868, p. 689, in a review of Dr. Seaton's *Handbook of Vaccination*, says: "Whatever risks there may be of syphilitic inoculation during vaccination, Dr. Seaton states that they are entirely risks of carelessness—risks, indeed, of such and so great carelessness that even as vaccination is too commonly performed in England they have an appreciable existence here. Neither our most experienced vaccinators nor syphilographers have ever met with a case in which syphilis has been communicated by the art of vaccination, and during the eight years, writes Dr. Seaton, in which there has been systematic inspection of public vaccination in England, some millions of vaccinations have been performed, but the inspectors have no knowledge of any such accident having occurred in any one instance (p. 338)."

The following proclamation was issued by Sir John Simon to Boards of Guardians during the small-pox epidemic, of virulence unequalled during the present century, of 1871. (To be found in the London *Times*, January 7th, 1871.)

PRIVY COUNCIL OFFICE,
MEDICAL DEPARTMENT, 8 RICHMOND TER.,
January 6th, 1871.

My Lords observe with regret that from time to time false and michievous statements are still spread among the poorer classes, with a view to excite hostility to vaccination, and tending to promote opposition to the law; but with reference to this subject, their lordships deem it almost superfluous to remind your Board that these statements, whether affecting to question the protecting power of vaccination or pretending to impute general ill consequences to practice, have again and again been utterly refuted by the most extensive and impartial inquiries, and that after seventy years' experience of vaccination, educated medical practitioners of every country of the world are practically unanimous in recommending its adoption.

I am, sir, your obedient servant,

(Signed) JOHN SIMON,
*Medical Officer of the Privy Council.*

Mr. Tebb then quotes the Report of the Select Committee on Vaccination, p. 3, a strong statement that there need be no apprehension that vaccination will impair health or communicate disease, and again on p. 387, in the appendix to the report, Sir John Simon reiterates this statement in another form, and further says: "Of the various alleged drawbacks to such great advantages, the present state of medical knowledge recognizes no single trace." From a pamphlet published in 1884, entitled *Facts Concerning Vaccination for Heads of Families*, revised by the Local Government Board and issued with their sanction, Mr. Tebb quotes similar statements, and concludes: "Against these statements I have submitted numerous fatal cases, disasters, terrible and widespread, of which vaccination has been the cause beyond possibility of denial or even of dispute, and where the observance of every proper precaution has exonerated the vaccinator from all blame, and revealed grave danger in the operation itself. The assertions which passed and have maintained the law have been thus shown to have been erroneous in theory and false in fact."

During the first two days of the delivery of Mr. Tebb's testimony and of his cross-examination, Lord Herschell, the Chairman, required the production of every authority for every statement made by Mr. Tebb. These he furnished to the very great disgust of Lord Herschell and the majority of the Commission. These consisted of medical journals, official reports, proclamations, correspondence, etc.

The production of the *Medical Observer*, published in the early part of the century, and of the suppressed report of the Royal College of Surgeons condemning vaccination, were examined with considerable interest and surprise, not to say dismay.

---

[The foregoing completes the testimony of Mr. Wm. Tebb. It is the condensation of many years' experience and effort to demonstrate the fallacies of vaccination. To this task he has consecrated most of his time and spent a large amount of money, currently believed to be $500,000! A short review of his life will be added, and the whole will be republished in book form.—Ed.]

